# Anxiety and depression and its correlates among undergraduate medical students in Nepal: A cross-sectional study

**DOI:** 10.1371/journal.pmen.0000560

**Published:** 2026-02-27

**Authors:** Milan Gaihre, Aashish Rana, Sandhya Niroula, Amit Kumar Sejuwal, Rhea Dhakal, Alisha Adhikari, Surya Devkota, Rakshya Shrestha

**Affiliations:** 1 Department of Internal Medicine, Institute of Medicine, Tribhuvan University Teaching Hospital (TUTH), Kathmandu, Nepal; 2 Institute of Medicine, Tribhuvan University, Kathmandu, Nepal; 3 Department of Internal Medicine, Jacobi Medical Center, Albert Einstein College of Medicine, New York, New York, United States of America; 4 Chitwan Medical College, Tribhuvan University, Chitwan, Nepal; 5 Department of Cardiology, Manmohan Cardiothoracic Vascular and Transplant Centre, Institute of Medicine, Kathmandu, Nepal; PLOS: Public Library of Science, UNITED KINGDOM OF GREAT BRITAIN AND NORTHERN IRELAND

## Abstract

Mental health problems like anxiety and depression among medical students are increasing. The academic stress, hectic duty, lack of personal and social life, financial hardship, etc., make them more vulnerable to poor mental health outcomes. This study aimed to determine the prevalence of anxiety and depression and their correlates among undergraduate medical students in Nepal. A web-based cross-sectional study was conducted in 2023 among 579 undergraduate MBBS and BDS students (≥18 years) enrolled in medical colleges under Tribhuvan University, Kathmandu University, Patan Academy of Health Sciences, and BP Koirala Institute of Health Sciences, Nepal. Symptoms of anxiety and depression were assessed using the validated Nepali version of the Hospital Anxiety and Depression Scale (HADS). Univariate analysis was carried out using frequency, percentage, mean, and standard deviation. Associations were examined using the chi-square test, followed by multivariate binary logistic regression. Statistical significance was set at p < 0.05 with a 95% confidence interval. The mean age of the participants was 21.0 ± 2.2 years and 53.7% were male. The prevalence of anxiety and depressive symptoms was 46.3% and 43.7%, respectively. Anxiety was significantly associated with female sex (AOR = 0.69, 95% CI: 0.49-0.98), Brahmin/Chhetri ethnic group (AOR = 0.64, 95% CI: 0.45-0.91), and presence of any disease (AOR = 8.51, 95% CI: 1.88-38.48). Depression was significantly associated with younger age (AOR = 1.96, 95% CI: 1.10-3.49), Brahmin/Chhetri ethnic group (AOR = 0.54, 95% CI: 0.38-0.78), father’s formal employment (AOR = 1.62, 95% CI: 1.13-2.33), being fourth year student (AOR = 3.25, 95% CI: 1.49-7.08) and daily consumption of green leafy vegetables (AOR = 0.66, 95% CI: 0.46-0.94). A high prevalence of anxiety and depressive symptoms was observed among undergraduate medical students in Nepal. These findings highlight the need for supportive academic environments and lifestyle-focused interventions to improve student well-being and strengthen the future healthcare workforce.

## Introduction

Mental disorders represent a leading cause of the global disease burden, affecting nearly one billion individuals worldwide [1]. Anxiety and depression are common mental health disorders affecting millions globally, with 301 million people living with an anxiety disorder and 280 million experiencing depression [2]. The prevalence of mental disorders has been increasing and continues to be a major contributor to the global disease burden. More than 80% of people with mental disorders live in low- and middle-income countries (LMICs) like Nepal [3]. The National Mental Health Survey, Nepal 2020 reported that nearly 10% of adults had experienced any mental disorders, and 6.5% had suicidal thoughts which is concerning [4]. Anxiety and depression cause health burden and may impair quality of life [5,6].

Medical students are particularly vulnerable to mental health issues such as anxiety and depression and often experience poorer psycho-social well-being compared to general population [7–9]. Risk factors include heavy academic pressure, financial stress, long hours of study, and high expectations [10,11]. Furthermore, students at this age are a critical developmental stage, putting them at a higher risk for psychiatric disorders, including major depressive disorder and anxiety, further compounding the mental health risks for medical students [12,13]. Medical students with mental disorders may experience substance abuse, suicidal thoughts, and have poor academic outcome [14,15].

The burden of mental disorders among medical students varies according to the geographic regions and education system contexts [16]. A scoping review of systematic reviews and meta-analyses published in Front Public Health (2026) showed a significant burden of mental health problems among medical students with the prevalence of depressive symptoms ranging from 18% to 50% and anxiety symptoms from 17% to 54% respectively [17], indicating that medical students are highly vulnerable to psychological distress. Adults of South Asian countries like Nepal are highly vulnerable to the burden of mental disorder and the cases of intentional self-harm are much prevalent in this age group [18]. A systematic review and meta-analysis in India, which included 19 studies found that about half of the undergraduate medical students experienced depressive symptoms [14]. Similarly, a study from Saudi Arabia reported that the prevalence of anxiety among undergraduate medical students ranged between 52.7% to 67% [19]. A cross-sectional study among 651 medical students and residents in Nepal, reported 45.3% had anxiety symptoms and 31% had depressive symptoms [20].

Despite the growing body of global evidence, the mental health landscape of medical students in Nepal remains under-explored, leaving a critical gap in the understanding of the prevalence and correlates of mental health issues in this population. Additionally, students from diverse socio-economic and cultural backgrounds come to pursue medical education in the medical colleges. Therefore, this study aimed to address this gap by assessing the prevalence of anxiety and depression among undergraduate medical students in Nepal through a web-based cross-sectional design. Considering the unique academic, social, and financial challenges faced by this population, gaining insight into their mental health status is crucial for developing effective and relevant interventions. The findings of this study are expected to guide the design of targeted strategies and support programs to promote the psychological well-being of future healthcare professionals in Nepal.

## Methods and materials

### Study design, setting, and participants

A web-based cross-sectional study was conducted among the undergraduate medical students pursuing Bachelor of Medicine and Bachelor of Surgery (MBBS) and Bachelor of Dental Surgery (BDS) under Tribhuvan University, Kathmandu University, Patan Academy of Health Sciences, and BP Koirala Institute of Health Sciences in Nepal. MBBS and BDS students aged ≥ 18 years studying in the selected medical colleges, enrolled from first year to final year were eligible to participate in this study.

The sample size was calculated using Cochran's formula [21], n = Z²pq/d², where n is the sample size, *Z* is the reliability coefficient at 95% confidence interval (1.96), p is the estimated proportion in the population (0.5),q = 1- p (0.5), and d is the maximum tolerable error (0.05). Although prior studies from Nepal have reported varying prevalence estimates of depression and anxiety among medical students, these estimates exhibit considerable heterogeneity across settings, academic years, institutions, and measurement tools. Thus, we conservatively used p = 0.5 to maximize the sample size and minimize the risk of under-powering the study. The calculated minimum sample size was 384. After adjusting for a 25% non-response rate, the final required sample size was 512. A consecutive sampling technique was used to recruit participants.

### Data collection procedure

Data were collected using a web-based survey administered through Google Forms from 10 June to 10 July 2023. The first page of the questionnaire contained information about the study objectives and procedures, followed by an electronic informed consent form. Only participants who provided informed consent were able to proceed to the questionnaire. Anonymity and confidentiality of responses were ensured.

Eight focal persons from the four selected institutions were identified and contacted by the principal investigator. The survey link was distributed through online platforms including Facebook, Whats App, and email in coordination with these focal persons. Participation was voluntary. To prevent duplicate responses, the “Limit to one response” option in Google Forms was enabled. In addition, the questionnaire included mandatory screening questions such as university name, affiliated medical college, program (MBBS/BDS), and year of study for the verification of eligible participants. Those students who did not respond to our Google Form could not be included in the study. There could be multiple reasons for this: not having internet access, lacking interest in participating, or missing to check their emails. Participants who did not provide electronic consent and forms with incomplete responses were considered as exclusion criteria for this study. However, in this study, all the participants had provided electronic consents and all the responses from the participants were complete. This might because undergraduate medical students belong to educated group and aware of importance and implication of such research.

### Measurement

The Hospital Anxiety and Depression Scale (HADS) was used to assess the anxiety and depressive symptoms of the undergraduate medical students [22]. We used a validated Nepali version HADS tool, having Cronbach’s alpha values of 0.76 and 0.68 for anxiety and depression sub-scales, respectively [23]. The scale consists of 14 items, with 7 items each for anxiety and depression. Each item is rated on a four-point scale (0–3), yielding possible scores ranging from 0 to 21 for both anxiety and depression. Based on the established cutoffs for the Nepali version [23], anxiety and depression were categorized as follows:

**Anxiety subscale**: No anxiety (0–7), borderline anxiety (89–10), and anxiety case (≥11)

**Depression subscale**: No depression (0–7), borderline depression (89–10), and depression case (≥11)

**Socio-demographic and health-related information:**

Socio-demographic variables included age, sex, ethnicity, current living status, parental occupational status, academic institution, academic program, and study level.

### Health-related variables assessed were

Medical history: presence of any disease such as hypertension, heart disease, Chronic Obstructive Pulmonary Disease (COPD), or diabetes (Yes/No), family history of hypertension, heart disease, COPD or diabetes (Yes/No),

Life style factors: checked lipid status in last 12 months (Yes/No), history of smoking in last 30 days (never, occasionally, always, past smoker), history of alcohol consumption in last 30 days (never, occasionally, always, past drinker), dietary type (veg/non-vegetarian), frequency of consumption of green leafy vegetables in last 7 days (never, once a week, 2/3 days a week, 4/5 days a week, daily, don't remember) and frequency of consumption of fruits in last 7 days (never, once a week, 2/3 days a week, 4/5 days a week, daily, don't remember).

Physical activity: Frequency of walking/cycling in the last 7 days and being physically active for at least 60 minutes a day in the last 7 days was also assessed.

The participants were asked to report their recently measured height and weight in the google form to determine the status of Body Mass Index (BMI).

Prior to the main data collection, the questionnaire was pretested among 10% of the estimated sample size to assess clarity, comprehension, and logical flow of the items. Internal consistency reliability was evaluated during the pretest, yielding Cronbach’s alpha values of 0.84 for the anxiety subscale and 0.77 for the depression subscale, indicating acceptable reliability. Data obtained from the pretest were excluded from the final analysis.

### Statistical analysis

The collected data were filtered, coded, and cross-checked using Microsoft Excel version 2019. Statistical analysis was performed using IBM SPSS Statistics version 21.

Descriptive statistics, including mean, frequency, and percentage, were used for univariate analysis. Normality was evaluated using both the Shapiro–Wilk test and the Kolmogorov–Smirnov test. The chi-square test was employed to examine associations between categorical independent and dependent variables. Variables with a p-value < 0.10 in the chi-square analysis were included in the multivariate logistic regression model [24]. Multivariate logistic regression was then conducted to assess the adjusted association between independent variables and the dependent variable, controlling for potential confounders. All analyses were performed at a 95% confidence interval, and a p-value < 0.05 was considered statistically significant.

### Ethical consideration

Ethical approval was taken from Ethical Review Board of the Nepal Health Research Council, Kathmandu, Nepal [Ref: 162–2021]. The study objectives and procedures were clearly outlined at the beginning of the survey. Electronic informed consent was obtained from all participants, who indicated their agreement by checking a consent box prior to participation in the Google Form. Participation in the study was entirely voluntary, and participants were informed of their right to withdraw from the study at any time without any consequences.

## Results

### Socio-demographic characteristics of the participants

The mean (SD) age of the participants was 21.0 ± 2.2 years. The majority of the respondents (53.7%) were male. More than half (57.6%) belonged to the Brahmin/Chhetri ethnic group. More than half (57.3%) of the respondents were studying under Tribhuvan University. Nearly 55% resided in hostels. The results are shown in Table 1.

**Table 1:** Socio-demographic characteristics of the participants (n = 579).

Characteristics	n (%)
**Age** Mean (SD)	21.0 ± 2.2
**Sex**	
Male	311 (53.7)
Female	268 (46.3)
**Ethnicity**	
Brahmin/Chhetri	334 (57.6)
Madhesi	152 (26.3)
Janajati	54 (9.3)
Dalit	12 (2.1)
Other	27 (4.7)
**Family type**	
Nuclear	444 (76.7)
Joint/ extended	135 (23.3)
**Father's occupation**	
Business	187 (32.3)
Governmental job	157 (27.1)
Agriculture	97 (16.8)
Non- governmental job	67 (11.6)
Foreign employment	50 (8.6)
Unemployed	11 (1.9)
Other	10 (1.7)
**Mother's occupation**	
House-maker	334 (57.7)
Governmental job	73 (12.6)
Business	63 (10.9)
Agriculture	32 (5.5)
Non-governmental job	32 (5.5)
Unemployed	27 (4.7)
Other	18 (3.1)
**Academic institution**	
Tribhuvan university	332 (57.3)
Kathmandu university	191 (33.0)
B.P. Koirala Institute of Health Sciences	36 (6.2)
Patan Academy of Health Sciences	20 (3.5)
**Academic program**	
MBBS	490 (84.6)
BDS	89 (15.4)
**Study year**	
1^st^ year	338 (58.4)
2^nd^ year	50 (8.6)
3^rd^ year	62 (10.7)
4^th^ year	54 (9.4)
Final year	75 (12.9)
**Living status**	
At hostel	316 (54.6)
With family	131 (22.6)
With friend	99 (17.1)
Alone	33 (5.7)

### Body Mass Index (BMI) and behavioral characteristics of the participants

Majority of the participants (68.7%) had a normal BMI index. More than half (54.2%) of the participants had a family history of heart disease, hypertension or diabetes. Most of the participants (91.0%) had never smoked, and 72.8% never consumed alcohol. Regarding dietary habits, 41.8% and 19.3% reported daily consumption of green leafy vegetables and fruits respectively. Very few (2.8%) reported having any disease such as hypertension, heart disease, COPD or diabetes. The findings are shown in Table 2.

**Table 2:** BMI, health history, and lifestyle of the participants (n = 579).

Characteristics	n (%)
**BMI category**	
Underweight	83 (14.3)
Normal	398 (68.7)
Overweight	75 (13.0)
Obese	23 (4.0)
**Family history of hypertension, heart disease, COPD or diabetes**	
Yes	314 (54.2)
No	265 (45.8)
**Presence of any disease such as hypertension, heart disease, COPD or diabetes**	
Yes	16 (2.8)
No	563 (97.2)
**Checked lipid profile (last 12 months)**	
Yes	79 (13.6)
No	500 (86.4)
**Lipid status (if checked)**	
Normal	70 (88.6)
Abnormal	9 (11.4)
**History of smoking (last 30 days)**	
Never	527 (91.0)
Occasionally	33 (5.7)
Past- smoker	13 (2.2)
Regular	6 (1.1)
**History of alcohol (last 30 days)**	
Never	421 (72.8)
Occasional	142 (24.5)
Past drinker	14 (2.4)
Regular	2 (0.3)
**Dietary type**	
Non-veg	512 (88.4)
Veg	67 (11.6)
**Consumption of green leafy vegetables in a week**	
Daily	242 (41.8)
4–5 days a week	138 (23.8)
2–3 days a week	137 (23.7)
Don't remember	46 (7.9)
Never	16 (2.8)
**Consumption of fruits in a week**	
Daily	112 (19.3)
4–5 days a week	101 (17.4)
2–3 days a week	157 (27.1)
Once a week	64 (11.1)
Don't remember	108 (18.7)
Never	37 (6.4)
**Frequency of physical activity (last 7 days)**	
**Never**	122 (21.1)
1 day	54 (9.3)
2 days	84 (14.5)
3 days	71 (12.3)
4 days	59 (10.2)
5 days	39 (6.7)
6 days	42 (7.3)
Daily	108 (18.7)
**Frequency of Walking or Cycling (last 7 days)**	
Never	180 (31.1)
1 days	26 (4.5)
2 days	21 (3.6)
3 days	24 (4.1)
4 days	18 (3.1)
5 days	33 (5.7)
6 days	109 (18.8)
Daily	168 (29.1)

### Anxiety and depressive symptoms among the participants

Nearly half (46.3%) of the respondents had symptoms of anxiety (Borderline anxiety = 26.3%, and Anxiety case = 20.0%) while 43.7% of them had symptoms of depression (Borderline depression = 33.2%, and Depression case = 10.5%). Details results are provided in Table 3.

**Table 3:** Anxiety and depressive symptoms among the participants (n = 579).

Characteristics	n (%)
**Anxiety category**	
No anxiety (0–7)	311 (53.7)
Borderline anxiety (8–10)	152 (26.3)
Anxiety case (≥11)	116 (20.0)
**Depression category**	
No depression (0–7)	326 (56.3)
Borderline depression (8–10)	192 (33.2)
Depression case (≥11)	61 (10.5)

### Socio-demographic, health-related, and lifestyle factors associated with symptoms of anxiety and depression

Several socio-demographic, health-related, and lifestyle factors showed a statistically significant association with symptoms of anxiety and depression based on the Pearson chi-square test. Sex (p-value = 0.030), ethnicity (p-value = 0.034), living status (p-value = 0.027), presence of any disease (p-value = 0.001), and physical activity (p-value = 0.017) showed significant association with anxiety symptoms. Whereas, age (p-value = 0.001), ethnicity (p-value = 0.004), father's occupation (p-value = 0.024), study year (p-value = 0.001), and consumption of green leafy vegetables (p-value = 0.020) were significantly associated with depressive symptoms. (Table 4)

**Table 4:** Factors associated with symptoms of anxiety and depression (n = 579).

Characteristics	Anxiety symptoms		Chi square value	p-value	Depression symptoms		Chi square value	p-value
	Yes (268; 46.3%)	No (311; 53.7%)			Yes (253; 43.7%)	No (326; 56.3%)		
**Age**								
21 or below	185 (48.6)	196 (51.4)	2.309	0.129	186 (48.8)	195 (51.2)	11.885	0.001
Above 21	83 (41.9)	115 (58.1)			67 (33.8)	131 (66.2)		
**Sex**								
Male	131 (42.1)	180 (57.9)	4.687	0.030	137 (44.1)	174 (55.9)	0.034	0.853
Female	137 (51.1)	131 (48.9)			116 (43.3)	152 (56.7)		
**Ethnicity**								
Brahmin/Chhetri	142 (42.5)	192 (57.5)	4.517	0.034	129 (38.6)	205 (61.4)	8.258	0.004
Other	126 (51.4)	119 (48.6)			124 (50.6)	121 (49.4)		
**Family type**								
Nuclear	202 (45.5)	242 (54.5)	0.479	0.489	191 (43.0)	253 (57.0)	0.356	0.551
Joint/ extended	66 (48.9)	69 (51.1)			62 (45.9)	73 (54.1)		
**Father's occupation**								
Gov/non-gov job	107 (47.8)	117 (52.2)	0.322	0.570	111 (49.6)	113 (50.4)	5.095	0.024
Other	161 (45.4)	194 (54.6)			142 (40.0)	213 (60.0)		
**Mother's occupation**								
House-maker	154 (46.1)	180 (53.9)	0.010	0.920	152 (45.5)	182 (54.5)	1.055	0.304
Other	114 (46.5)	131 (53.5)			101 (41.2)	144 (58.8)		
**Academic program**								
MBBS	228 (46.5)	262 (53.5)	0.076	0.782	212 (43.3)	278 (56.7)	0.240	0.624
BDS	40 (44.9)	49 (55.1)			41 (46.1)	48 (53.9)		
**Study year**								
1^st^ year	157 (46.4)	181 (53.6)	5.207	0.267	157 (46.4)	181 (53.6)	17.840	0.001
2^nd^ year	28 (56.0)	22 (44.0)			27 (54.0)	23 (46.0)		
3^rd^ year	32 (51.6)	30 (48.4)			25 (40.3)	37 (59.7)		
4^th^ year	20 (37.0)	34 (63.0)			27 (50.0)	27 (50.0)		
Final year	31 (41.3)	44 (58.7)			17 (22.7)	58 (77.3)		
**University**								
Tribhuvan university	151 (45.5)	181 (54.5)	0.203	0.653	134 (40.4)	198 (59.6)	0.351	0.617
Other	117 (47.4)	130 (52.6)			119 (48.2)	128 (51.8)		
**Living status**								
Alone or friend	50 (37.9)	82 (62.1)	4.862	0.027	48 (36.4)	84 (63.6)	3.736	0.053
Hostel or family	218 (48.8)	229 (51.2)			205 (45.9)	242 (54.1)		
**BMI**								
Underweight	42 (50.6)	41 (49.4)	0.866	0.649	38 (45.8)	45 (54.2)	0.285	0.867
Normal	183 (46.0)	215 (54.0)			174 (43.7)	224 (56.3)		
Overweight/obese	43 (43.9)	55 (56.1)			41 (41.8)	57 (58.2)		
**Family history of hypertension, heart disease, CODP or diabetes**								
Yes	153 (48.7)	161 (51.3)	1.642	0.200	136 (43.3)	178 (56.7)	0.041	0.839
No	115 (43.4)	150 (43.4)			117 (44.2)	148 (55.8)		
**Presence of any disease**								
Yes	14 (87.5)	2 (12.5)	11.242	0.001	9 (56.3)	7 (43.8)	1.054	0.305
No	254 (45.1)	309 (54.9)			244 (43.3)	319 (56.7)		
**History of smoking (last 30 days)**								
Never smoked	244 (46.3)	283 (53.7)	0.000	0.984	233 (44.2)	294 (55.8)	0.636	0.425
Smoked	24 (46.2)	28 (53.8)			20 (38.5)	32 (61.5)		
**History of drinking (last 30 days)**								
Never drank	195 (46.3)	226 (53.7)	0.001	0.980	182 (43.2)	239 (56.8)	0.136	0.712
Drank	73 (46.2)	85 (53.8)			71 (44.9)	87 (55.1)		
**Physical activity at least 5 days a week (last 7 days)**								
Yes	74 (39.2)	115 (60.8)	5.743	0.017	73 (38.6)	116 (61.4)	2.934	0.087
No	194 (49.7)	196 (50.3)			180 (46.2)	210 (53.8)		
**Walking or cycling at least 5 days a week (last 7 days)**								
Yes	139 (44.8)	171 (55.2)	0.563	0.453	129 (41.6)	181 (58.4)	1.177	0.278
No	129 (48.0)	140 (52.0)			124 (46.1)	145 (53.9)		
**Dietary habit**								
Non-veg	237 (46.3)	275 (53.7)	0.000	0.997	227 (44.3)	285 (55.7)	0.736	0.391
Veg	31 (46.3)	36 (53.7)			26 (38.8)	41 (61.2)		
**Consumed fruits everyday (last 7 days)**								
Yes	46 (41.1)	66 (58.9)	1.519	0.218	45 (40.2)	67 (59.8)	0.698	0.403
No	222 (47.5)	245 (52.5)			208 (44.5)	259 (55.5)		
**Consumed green leafy vegetable everyday (last 7 days)**								
Yes	102 (42.1)	140 (57.9)	2.863	0.091	92 (38.0)	150 (62.0)	5.451	0.020
No	166 (49.3)	171 (50.7)			161 (47.8)	176 (52.2)		

### Multivariate binary logistic regression for factors associated with symptoms of anxiety and depression

The multivariate binary logistic regression analyses showed that female sex (AOR = 0.69, 95% CI: 0.49-0.98), Brahmin/Chhetri ethnic group (AOR = 0.64, 95% CI: 0.45-0.91), and presence of any disease (AOR = 8.51, 95% CI: 1.88-38.48) were significantly associated with symptoms of anxiety. (Table 5)

**Table 5:** Multivariate binary logistic regression for factors associated with symptoms of anxiety.

Variables	AOR at 95% CI	p-value
**Sex**		
Male	0.69 (0.49-0.98)	0.043*
Female	Ref	
**Ethnicity**		
Brahmin/Chhetri	0.64 (0.45-0.91)	0.013*
Other	Ref	
**Living status**		
Alone or friend	0.70 (0.46-1.07)	0.102
Hostel or family	Ref	
**Presence of any disease**		
Yes	8.51 (1.88-38.48)	0.005*
No	Ref	
**Physical activity at least 5 days a week (last 7 days)**		
Yes	0.75 (0.52-1.09)	0.143
No	Ref	
**Consumption of green leafy vegetable everyday (last 7 days)**		
Yes	0.75 (0.53-1.06)	0.114
No	Ref	

Ref: reference

AOR: Adjusted Odds Ratio

*: statistically significant at p-value < 0.05

Younger age (AOR = 1.96, 95% CI: 1.10-3.49), Brahmin/Chhetri ethnic group (AOR = 0.54, 95% CI: 0.38-0.78), those whose fathers are engaged formal government or non-governmental jobs (AOR = 1.62, 95% CI: 1.13-2.33), being fourth year student (AOR = 3.25, 95% CI: 1.49-7.08) and daily consumption of green leafy vegetables (AOR = 0.66, 95% CI: 0.46-0.94) were significantly associated with symptoms of depression. (Table 6)

**Table 6:** Multivariate binary logistic regression for factors associated with symptoms of depression.

Characteristics	AOR at 95% CI	p-value
**Age**		
≤ 21 years	1.96 (1.10-3.49)	0.021*
Above 21 years	Ref	
**Ethnicity**		
Brahmin/Chhetri	0.54 (0.38-0.78)	0.001*
Other	Ref	
**Father's occupation**		
Gov/non-gov job	1.62 (1.13-2.33)	0.008*
Other	Ref	
**Study year**		
1^st^ year	1.40 (0.63-3.12)	0.020*
2^nd^ year	2.37 (0.96-5.84)	
3^rd^ year	1.69 (0.76-3.76)	
4^th^ year	3.25 (1.49-7.08)	
Final year	Ref	
**Living status**		
Alone or friend	0.79 (0.53-1.24)	0.315
Hostel or family	Ref	
**Physical activity at least 5 days a week (last 7 days)**		
Yes	0.79 (0.54-1.15)	0.230
No	Ref	
**Consumption of green leafy vegetable everyday (last 7 days)**		
Yes	0.66 (0.46-0.94)	0.022*
No	Ref	

Ref: reference

AOR: Adjusted Odds Ratio

*: statistically significant at p-value < 0.05

## Discussion

This web-based study conducted among 579 undergraduate medical students in Nepal demonstrates a substantial mental health burden in this population, with nearly half of the participants reporting symptoms of anxiety (46.3%) and depression (43.7%).

The prevalence of anxiety observed in this study is comparable to findings from earlier studies conducted in Nepal (45.3%) [20], and Germany (49.0%) [25]. However, a higher prevalence of anxiety among medical students has been reported in studies from Nepal (59.3%) [26], Egypt (73.0%) [27], and Saudi Arabia (55.4%) [28]. In contrast, relatively lower levels of anxiety were documented in studies conducted in Nepal (5.8%) [29], and India (20.0%) [30]. Regarding the prevalence of depressive symptoms, the finding of this study is comparable to studies carried out in Nepal (44.0%) [26], and Ethiopia (51.3%) [31]. Meanwhile, studies carried out in Palestine (56.6%) [32], and Egypt (65.0%) [27] reported higher rates of depressive symptoms among medical students. Meanwhile, other studies conducted in Nepal (29.2%) [29], and India (13.9%) [30] have revealed relatively lower prevalence of depression. The observed variation in prevalence of anxiety and depression across studies might be due to difference in sample size, assessment tools used to measure mental health outcomes, and existing socio-cultural difference among the countries.

This study revealed that age was statistically significant with depressive symptoms. Students aged 21 years or below had higher odds of having depressive symptoms than older age groups. This is supported by the findings from the previous studies carried out in Saudi Arabia [28], and Spain [33]. However, studies carried out in India [34], and Turkey [35] reported higher odds of depressive symptoms among older age groups. In this study, higher odds of depressive symptoms among younger aged group might be because at the initial phase of medical college, the students have to face lots of challenges in building social connections, adjusting to a new institutional environment, as well as academic-related stress. This can contribute to feelings of isolation, low morale, and reduced coping capacity, putting them at high risk of psychological burden.

Similarly, this study revealed a significant association between sex and anxiety symptoms. Consistent with other studies in Ethiopia [31] and India [36], females had higher odds of having anxiety symptoms as compared to males. A nationally representative mental health survey by the WHO also concluded that women had higher mood and anxiety disorders as compared to men [37]. This gender disparity may be attributed to both biological and psychological differences, as males and females tend to respond differently to stressors [38]. Moreover, the menstrual cycle and hormonal fluctuations in females can significantly influence the psychological stress response, potentially increasing vulnerability to anxiety [38,39]. Compounded by personal traits, gender inequality, and socio-cultural stigma, these factors may further intensify the mental health burden among women in Nepal.

Similarly, ethnicity was also one of the factors associated with mental health burden. This study found that Brahmin/Chhetri had lower odds of having anxiety and depressive symptoms than others. The previous studies also suggest that mental disorders vary across ethnic groups [40–42]. A secondary analysis of data from Nepal Demographic and Health Survey 2022 showed that Dalits had higher odds of having poor mental health outcomes than other ethnic groups [43]. This disparity may be attributed to cultural practices, stigma, and social norms that influence socioeconomic status and access to health services, thus affecting mental health status [44,45]. Those who belonged to an ethnic group other than Brahmin/Chhetri may have social stigma and discrimination, poor education and income instability in the family, all of which increase their vulnerability to poor mental health outcome.

In this study, father's occupational status was found to be one of the determinants of depression. Those whose father had government or non-government jobs had 1.6 times higher odds of experiencing symptoms of depression than others. However, several studies have revealed that unemployment and the risky occupation of parents are associated with poor mental health status [46,47]. The higher odds of depressive symptoms observed among students whose fathers were formally employed may be attributed to reduced parental availability and support due to busy schedule and workload. Insufficient parental involvement and support during critical developmental stages can adversely affect the psychological, emotional, and overall growth of children, thereby increasing their vulnerability to depressive symptoms [48].

This study revealed that the study year was significantly associated with depressive symptoms. This finding coincides with the findings of previous studies [49–51]. Our study found that the students in the fourth year had higher odds of developing symptoms of depression. Meanwhile, the previous studies suggest that the prevalence of depression varies across the study years [11,29,31,51]. This is due to teaching and learning related stressors [11]. Academic burden, frequency of examinations, curriculum, performance of students, financial burden, lack of social life, etc., are common stressors among medical students [51–53]. Incorporating stress management workshops, mentorship schemes, periodic curriculum reviews to balance academic workload and time management training into the curriculum can help students develop effective coping strategies to reduce the risk of depression among medical students.

Also, this study found a significant association between the presence of any disease such as hypertension, COPD, Type 2 diabetes and heart disease and symptoms of anxiety. Students with presence of such diseases were more likely to have symptoms of anxiety. This finding aligns with previous studies, which have consistently shown that individuals with chronic illnesses and co-morbidities are at high risk of experiencing psychological distress [54,55]. A study in U.S by Sareen et al. found that physical health conditions like cardiovascular disease, tuberculosis, diabetes, and kidney disease were associated with high rates of anxiety disorders [56]. Frequent medical visits, treatment cost, and lifestyle modification for the management of chronic diseases can act as a stressor contributing to emotional strain.

In this study, those who reported daily consumption of green leafy vegetables were less 0.6 times likely to have depressive symptoms. Several studies have shown that healthier eating habits, particularly regular intake of fruits and vegetables, are linked to lower levels of anxiety and depression [57–60]. This association may be due to the antioxidants, vitamins, and minerals in these foods that help reduce oxidative stress involved in depression [61–63].

### Strengths

One of the key strengths of this study is the large sample size, which included undergraduate students from multiple medical colleges. A validated Nepali version of the Hospital Anxiety and Depression Scale (HADS) was used to reliably assess anxiety and depressive symptoms. Additionally, the web-based survey design ensured confidentiality and privacy, encouraging honest responses on sensitive behavioral risk factors such as alcohol consumption and smoking.

### Limitations

As this study was conducted as a web-based survey, it may be subject to recall bias, may not fully represent the entire population, and limits the generalizability of the findings. Important factors such as past academic failure, academic satisfaction, and participation in recreational or extracurricular activities were not included as predictor variables. Additionally, we did not assess the influence of family support, social support, or students’ coping skills. Also, this study did not assess the history of mental illness, use of psychiatric drugs, long-term medication and health condition other than COPD, heart disease, and hypertension, which could influence the study outcome. Importantly, anxiety and depression were assessed using the HADS, which is a screening instrument rather than a diagnostic tool. Thus, scores above the established cut-offs indicate probable cases of anxiety or depression but do not equate to confirmed clinical diagnoses. Consequently, the reported proportions should be interpreted as the prevalence of elevated anxiety and depressive symptoms, rather than definitive rates of clinically diagnosed disorders. Another limitation is the study's cross-sectional design, which precludes establishing causal relationships between variables.

## Conclusion

This study highlights a substantial burden of anxiety and depressive symptoms among undergraduate medical students in Nepal, with nearly half of the participants reporting symptoms of anxiety (46.3%) and depression (43.7%). Age, sex, ethnicity, father's occupational status, year of study, presence of any chronic illnesses, and dietary practice were determinants of anxiety and depressive symptoms. The findings indicate that both socio-demographic and academic-related factors contribute to the mental health burden among medical students. These findings underscore the need for institutional strategies such as routine mental health screening, counselling services, stress management programs, promotion of healthy lifestyles and flexible work policies for parents, and ensuring a supportive learning environment, to effectively address mental health concerns, improve students’ well-being and foster a healthier and more resilient future healthcare workforce.

## Supporting information

S1 Data Dataset used for analysis. (XLSX)

## References

[1] Nearly one billion people have a mental disorder: WHO. UN News. https://news.un.org/en/story/2022/06/1120682. 2022.

[2] Mental disorders. https://www.who.int/news-room/fact-sheets/detail/mental-disorders 2023 October 1.

[3] Mudunna C, Weerasinghe M, Tran T, Antoniades J, Romero L, Chandradasa M, et al. Nature, prevalence and determinants of mental health problems experienced by adolescents in south Asia: a systematic review. Lancet Reg Health Southeast Asia. 2025;33:100532. doi: 10.1016/j.lansea.2025.100532 39902294 PMC11788858

[4] NHRC. Report of National Mental Health Survey 2020. Nepal Health Research Council, Government of Nepal. 2021. https://nhrc.gov.np/wp-content/uploads/2022/10/National-Mental-Health-Survey-Report2020.pdf

[5] Shanafelt TD, West C, Zhao X, Novotny P, Kolars J, Habermann T, et al. Relationship between increased personal well-being and enhanced empathy among internal medicine residents. J Gen Intern Med. 2005;20(7):559–64. doi: 10.1111/j.1525-1497.2005.0108.x 16050855 PMC1490167

[6] Dyrbye LN, Harper W, Moutier C, Durning SJ, Power DV, Massie FS, et al. A multi-institutional study exploring the impact of positive mental health on medical students’ professionalism in an era of high burnout. Acad Med. 2012;87(8):1024–31. doi: 10.1097/ACM.0b013e31825cfa35 22722352

[7] Pacheco JP, Giacomin HT, Tam WW, Ribeiro TB, Arab C, Bezerra IM, et al. Mental health problems among medical students in Brazil: a systematic review and meta-analysis. Braz J Psychiatry. 2017;39(4):369–78. doi: 10.1590/1516-4446-2017-2223 28876408 PMC7111407

[8] Pagnin D, de Queiroz V. Comparison of quality of life between medical students and young general populations. Educ Health (Abingdon). 2015;28(3):209–12. doi: 10.4103/1357-6283.178599 26996647

[9] Dyrbye LN, West CP, Satele D, Boone S, Tan L, Sloan J, et al. Burnout among U.S. medical students, residents, and early career physicians relative to the general U.S. population. Acad Med. 2014;89(3):443–51. doi: 10.1097/ACM.0000000000000134 24448053

[10] Al-Khani AM, Sarhandi MI, Zaghloul MS, Ewid M, Saquib N. A cross-sectional survey on sleep quality, mental health, and academic performance among medical students in Saudi Arabia. BMC Res Notes. 2019;12(1):665. doi: 10.1186/s13104-019-4713-2 31639038 PMC6802108

[11] Agyapong-Opoku G, Agyapong B, Obuobi-Donkor G, Eboreime E. Depression and Anxiety among Undergraduate Health Science Students: A Scoping Review of the Literature. Behav Sci (Basel). 2023;13(12):1002. doi: 10.3390/bs13121002 38131858 PMC10740739

[12] Patten SB. Major depression prevalence in Calgary. Can J Psychiatry. 2000;45(10):923–6. doi: 10.1177/070674370004501008 11190362

[13] Stewart-Brown S, Evans J, Patterson J, Petersen S, Doll H, Balding J, et al. The health of students in institutes of higher education: an important and neglected public health problem?. J Public Health Med. 2000;22(4):492–9. doi: 10.1093/pubmed/22.4.492 11192277

[14] Dutta G, Rajendran N, Kumar T, Varthya SB, Rajendran V. Prevalence of Depression Among Undergraduate Medical Students in India: A Systemic Review and Meta-Analysis. Cureus. 2023;15(1):e33590. doi: 10.7759/cureus.33590 36779123 PMC9910033

[15] Mhata NT, Ntlantsana V, Tomita AM, Mwambene K, Saloojee S. Prevalence of depression, anxiety and burnout in medical students at the University of Namibia. S Afr J Psychiatr. 2023;29:2044. doi: 10.4102/sajpsychiatry.v29i0.2044 37292521 PMC10244924

[16] Vagiri R, Mohlabe K, Mailula L, Nhubunga F, Maepa M, Mphasha M, et al. Exploring Anxiety and Depression Among Medical Undergraduates in South Africa: A Cross-Sectional Survey. Healthcare (Basel). 2025;13(6):649. doi: 10.3390/healthcare13060649 40150499 PMC11941862

[17] Agyapong-Opoku N, Agyapong-Opoku F, Agyapong B, Greenshaw AJ. Anxiety and depressive symptoms among medical students-A scoping review of systematic reviews and meta-analyses. Front Public Health. 2026;13:1710333. doi: 10.3389/fpubh.2025.1710333 41551283 PMC12807908

[18] Vidyasagaran AL, McDaid D, Faisal MR, Nasir M, Muliyala KP, Thekkumkara S, et al. Prevalence of mental disorders in South Asia: A systematic review of reviews. Glob Ment Health (Camb). 2023;10:e78. doi: 10.1017/gmh.2023.72 38161740 PMC10755414

[19] Dabbagh R, Alwatban L, Alrubaiaan M, Alharbi S, Aldahkil S, AlMuteb M, et al. Depression, stress, anxiety and burnout among undergraduate and postgraduate medical trainees in Saudi Arabia over two decades: A systematic review. Med Teach. 2023 May;45(5):499–509. doi: 10.1080/0142159X.2022.2139669 36355388

[20] Pokhrel NB, Khadayat R, Tulachan P. Depression, anxiety, and burnout among medical students and residents of a medical school in Nepal: a cross-sectional study. BMC Psychiatry. 2020;20(1):298. doi: 10.1186/s12888-020-02645-6 32539732 PMC7294639

[21] Cochran WG. Sampling Techniques, Third Edition.

[22] Snaith RP. The Hospital Anxiety And Depression Scale. Health Qual Life Outcomes. 2003;1:29. doi: 10.1186/1477-7525-1-29 12914662 PMC183845

[23] Risal A, Manandhar K, Linde M, Koju R, Steiner TJ, Holen A. Reliability and Validity of a Nepali-language Version of the Hospital Anxiety and Depression Scale (HADS). Kathmandu Univ Med J (KUMJ). 2015;13(50):115–24. doi: 10.3126/kumj.v13i2.16783 26657079

[24] Paudel K, Adhikari TB, Khanal P, Bhatta R, Paudel R, Bhusal S, et al. Sleep quality and its correlates among undergraduate medical students in Nepal: A cross-sectional study. PLOS Glob Public Health. 2022;2(2):e0000012. doi: 10.1371/journal.pgph.0000012 36962248 PMC10021869

[25] Akhtar M, Herwig BK, Faize FA. Depression and Anxiety among International Medical Students in Germany: The Predictive Role of Coping Styles. J Pak Med Assoc. 2019;69(2):230–4. 30804589

[26] Shah P, Sapkota A, Chhetri A. Depression, Anxiety, and Stress among First-year Medical Students in a Tertiary Care Hospital: A Descriptive Cross-sectional Study. JNMA J Nepal Med Assoc. 2021;59(236):346–9. doi: 10.31729/jnma.6420 34508520 PMC8369603

[27] Fawzy M, Hamed SA. Prevalence of psychological stress, depression and anxiety among medical students in Egypt. Psychiatry Res. 2017;255:186–94. doi: 10.1016/j.psychres.2017.05.027 28575777

[28] Al-Garni AM, Shati AA, Almonawar NA, Alamri GM, Alasmre LA, Saad TN, et al. Prevalence of depression, anxiety, and stress among students enrolled at King Khalid University: a cross-sectional study. BMC Public Health. 2025;25(1):354. doi: 10.1186/s12889-025-21277-7 39875847 PMC11773868

[29] Adhikari A, Dutta A, Sapkota S, Chapagain A, Aryal A, Pradhan A. Prevalence of poor mental health among medical students in Nepal: a cross-sectional study. BMC Med Educ. 2017;17(1):232. doi: 10.1186/s12909-017-1083-0 29183315 PMC5704530

[30] Arun P, Ramamurthy P, Thilakan P. Indian Medical Students with Depression, Anxiety, and Suicidal Behavior: Why Do They Not Seek Treatment?. Indian J Psychol Med. 2022;44(1):10–6. doi: 10.1177/0253717620982326 35509655 PMC9022918

[31] Kebede MA, Anbessie B, Ayano G. Prevalence and predictors of depression and anxiety among medical students in Addis Ababa, Ethiopia. Int J Ment Health Syst. 2019;13:30. doi: 10.1186/s13033-019-0287-6 31080499 PMC6501290

[32] Shawahna R, Hattab S, Al-Shafei R, Tab’ouni M. Prevalence and factors associated with depressive and anxiety symptoms among Palestinian medical students. BMC Psychiatry. 2020;20(1):244. doi: 10.1186/s12888-020-02658-1 32429889 PMC7236464

[33] Gijón Puerta J, Galván Malagón MC, Khaled Gijón M, Lizarte Simón EJ. Levels of Stress, Anxiety, and Depression in University Students from Spain and Costa Rica during Periods of Confinement and Virtual Learning. Education Sciences. 2022;12(10):660. doi: 10.3390/educsci12100660

[34] Naushad S, Farooqui W, Sharma S, Rani M, Singh R, Verma S. Study of proportion and determinants of depression among college students in Mangalore city. Niger Med J. 2014;55(2):156–60. doi: 10.4103/0300-1652.129657 24791051 PMC4003720

[35] Bostanci M, Ozdel O, Oguzhanoglu NK, Ozdel L, Ergin A, Ergin N, et al. Depressive symptomatology among university students in Denizli, Turkey: prevalence and sociodemographic correlates. Croat Med J. 2005;46(1):96–100. 15726682

[36] Iqbal S, Gupta S, Venkatarao E. Stress, anxiety and depression among medical undergraduate students and their socio-demographic correlates. Indian J Med Res. 2015;141(3):354–7. doi: 10.4103/0971-5916.156571 25963497 PMC4442334

[37] Seedat S, Scott KM, Angermeyer MC, Berglund P, Bromet EJ, Brugha TS, et al. Cross-national associations between gender and mental disorders in the World Health Organization World Mental Health Surveys. Arch Gen Psychiatry. 2009;66(7):785–95. doi: 10.1001/archgenpsychiatry.2009.36 19581570 PMC2810067

[38] Kajantie E, Phillips DIW. The effects of sex and hormonal status on the physiological response to acute psychosocial stress. Psychoneuroendocrinology. 2006;31(2):151–78. doi: 10.1016/j.psyneuen.2005.07.002 16139959

[39] Lindheim SR, Legro RS, Bernstein L, Stanczyk FZ, Vijod MA, Presser SC, et al. Behavioral stress responses in premenopausal and postmenopausal women and the effects of estrogen. Am J Obstet Gynecol. 1992;167(6):1831–6. doi: 10.1016/0002-9378(92)91783-7 1471706

[40] Jager PD, Suliman S, Seedat S. Role of ethnicity in social anxiety disorder: A cross-sectional survey among health science students. World J Clin Cases. 2014;2(7):265–71. doi: 10.12998/wjcc.v2.i7.265 25032201 PMC4097153

[41] MacIntyre MM, Zare M, Williams MT. Anxiety-Related Disorders in the Context of Racism. Curr Psychiatry Rep. 2023;25(2):31–43. doi: 10.1007/s11920-022-01408-2 36645562 PMC9841144

[42] Eylem O, de Wit L, van Straten A, Steubl L, Melissourgaki Z, Danışman GT, et al. Stigma for common mental disorders in racial minorities and majorities a systematic review and meta-analysis. BMC Public Health. 2020;20(1):879. doi: 10.1186/s12889-020-08964-3 32513215 PMC7278062

[43] Pandey AR, Adhikari B, Bista B, Lamichhane B, Joshi D, K C SP, et al. Prevalence, determinants and care-seeking behaviour for anxiety and depression in Nepalese population: a secondary analysis of data from Nepal Demographic and Health Survey 2022. BMJ Open. 2024;14(8):e078582. doi: 10.1136/bmjopen-2023-078582 39107021 PMC11308907

[44] Kohrt BA, Speckman RA, Kunz RD, Baldwin JL, Upadhaya N, Acharya NR, et al. Culture in psychiatric epidemiology: using ethnography and multiple mediator models to assess the relationship of caste with depression and anxiety in Nepal. Ann Hum Biol. 2009;36(3):261–80. doi: 10.1080/03014460902839194 19381985 PMC3907946

[45] Kirkbride JB, Anglin DM, Colman I, Dykxhoorn J, Jones PB, Patalay P, et al. The social determinants of mental health and disorder: evidence, prevention and recommendations. World Psychiatry. 2024;23(1):58–90. doi: 10.1002/wps.21160 38214615 PMC10786006

[46] Li J, Loerbroks A, Siegrist J. Effort-reward Imbalance at Work, Parental Support, and Suicidal Ideation in Adolescents: A Cross-sectional Study from Chinese Dual-earner Families. Saf Health Work. 2017;8(1):77–83. doi: 10.1016/j.shaw.2016.09.003 28344844 PMC5355538

[47] Maggi S, Ostry A, Tansey J, Dunn J, Hershler R, Chen L, et al. Paternal psychosocial work conditions and mental health outcomes: a case-control study. BMC Public Health. 2008;8:104. doi: 10.1186/1471-2458-8-104 18377651 PMC2358891

[48] Fuller AE, Shahidi FV, Comeau J, Wang L, Wahi G, Dunn JR. Parental employment quality and the mental health and school performance of children and youth. J Epidemiol Community Health. 2025. 10.1136/jech-2024-223366 PMC12322398 40037834

[49] Taple BJ, Chapman R, Schalet BD, Brower R, Griffith JW. The Impact of Education on Depression Assessment: Differential Item Functioning Analysis. Assessment. 2022;29(2):272–84. doi: 10.1177/1073191120971357 33218257 PMC9060911

[50] Moutinho ILD, Maddalena N de CP, Roland RK, Lucchetti ALG, Tibiriçá SHC, Ezequiel O da S, et al. Depression, stress and anxiety in medical students: A cross-sectional comparison between students from different semesters. Rev Assoc Med Bras (1992). 2017;63(1):21–8. doi: 10.1590/1806-9282.63.01.21 28225885

[51] Pukas L, Rabkow N, Keuch L, Ehring E, Fuchs S, Stoevesandt D, et al. Prevalence and predictive factors for depressive symptoms among medical students in Germany - a cross-sectional study. GMS J Med Educ. 2022;39(1):Doc13. doi: 10.3205/zma001534 35368844 PMC8953191

[52] Sreeramareddy CT, Shankar PR, Binu VS, Mukhopadhyay C, Ray B, Menezes RG. Psychological morbidity, sources of stress and coping strategies among undergraduate medical students of Nepal. BMC Med Educ. 2007;7:26. doi: 10.1186/1472-6920-7-26 17678553 PMC1951961

[53] Al-Khlaiwi T, Habib SS, Akram A, Al-Khliwi H, Habib SM. Comparison of depression, anxiety, and stress between public and private university medical students. J Family Med Prim Care. 2023;12(6):1092–8. doi: 10.4103/jfmpc.jfmpc_1719_22 37636173 PMC10451576

[54] Duko B, Gebeyehu A, Ayano G. Prevalence and correlates of depression and anxiety among patients with tuberculosis at WolaitaSodo University Hospital and Sodo Health Center, WolaitaSodo, South Ethiopia, cross sectional study. BMC Psychiatry. 2015;15:214. 26370894 10.1186/s12888-015-0598-3 PMC4570612

[55] Walker ER, McGee RE, Druss BG. Mortality in mental disorders and global disease burden implications. JAMA Psychiatry. 2015;72(4):334–41. 25671328 10.1001/jamapsychiatry.2014.2502 PMC4461039

[56] Sareen J, Cox BJ, Clara I, Asmundson GJG. The relationship between anxiety disorders and physical disorders in the U.S. National Comorbidity Survey. Depress Anxiety. 2005;21(4):193–202. doi: 10.1002/da.20072 16075453

[57] Fismen A-S, Aarø LE, Thorsteinsson E, Ojala K, Samdal O, Helleve A, et al. Associations between eating habits and mental health among adolescents in five nordic countries: a cross-sectional survey. BMC Public Health. 2024;24(1):2640. doi: 10.1186/s12889-024-20084-w 39334065 PMC11438251

[58] Gibson-Smith D, Bot M, Brouwer IA, Visser M, Giltay EJ, Penninx BWJH. Association of food groups with depression and anxiety disorders. Eur J Nutr. 2020;59(2):767–78. doi: 10.1007/s00394-019-01943-4 30945032 PMC7058560

[59] Ju S-Y, Park YK. Low fruit and vegetable intake is associated with depression among Korean adults in data from the 2014 Korea National Health and Nutrition Examination Survey. J Health Popul Nutr. 2019;38(1):39. doi: 10.1186/s41043-019-0204-2 31796113 PMC6892133

[60] McMartin SE, Jacka FN, Colman I. The association between fruit and vegetable consumption and mental health disorders: evidence from five waves of a national survey of Canadians. Prev Med. 2013;56(3–4):225–30. doi: 10.1016/j.ypmed.2012.12.016 23295173

[61] Apostolakopoulou XA, Petinaki E, Kapsoritakis AN, Bonotis K. A Narrative Review of the Association Between Healthy Dietary Patterns and Depression. Cureus. 2024;16(5):e60920. doi: 10.7759/cureus.60920 38910729 PMC11193411

[62] Rahaman MM, Hossain R, Herrera-Bravo J, Islam MT, Atolani O, Adeyemi OS, et al. Natural antioxidants from some fruits, seeds, foods, natural products, and associated health benefits: An update. Food Sci Nutr. 2023;11(4):1657–70. doi: 10.1002/fsn3.3217 37051367 PMC10084981

[63] Wang H, Jin M, Xie M, Yang Y, Xue F, Li W, et al. Protective role of antioxidant supplementation for depression and anxiety: A meta-analysis of randomized clinical trials. J Affect Disord. 2023;323:264–79. doi: 10.1016/j.jad.2022.11.072 36442656

